# Mechanisms Underlying Tolerance after Long-Term Benzodiazepine Use: A Future for Subtype-Selective GABA_A_ Receptor Modulators?

**DOI:** 10.1155/2012/416864

**Published:** 2012-03-29

**Authors:** Christiaan H. Vinkers, Berend Olivier

**Affiliations:** ^1^Division of Pharmacology, Utrecht Institute for Pharmaceutical Sciences and Rudolf Magnus Institute of Neuroscience, Utrecht University, Universiteitsweg 99, 3584CG Utrecht, The Netherlands; ^2^Department of Psychiatry, Rudolf Magnus Institute of Neuroscience, University Medical Center Utrecht, Utrecht, The Netherlands; ^3^Department of Psychiatry, Yale University School of Medicine, New Haven, CT, USA

## Abstract

Despite decades of basic and clinical research, our understanding of how benzodiazepines tend to lose their efficacy over time (tolerance) is at least incomplete. In appears that tolerance develops relatively quickly for the sedative and anticonvulsant actions of benzodiazepines, whereas tolerance to anxiolytic and amnesic effects probably does not develop at all. In light of this evidence, we review the current evidence for the neuroadaptive mechanisms underlying benzodiazepine tolerance, including changes of (i) the GABA_A_ receptor (subunit expression and receptor coupling), (ii) intracellular changes stemming from transcriptional and neurotrophic factors, (iii) ionotropic glutamate receptors, (iv) other neurotransmitters (serotonin, dopamine, and acetylcholine systems), and (v) the neurosteroid system. From the large variance in the studies, it appears that either different (simultaneous) tolerance mechanisms occur depending on the benzodiazepine effect, or that the tolerance-inducing mechanism depends on the activated GABA_A_ receptor subtypes. Importantly, there is no convincing evidence that tolerance occurs with *α* subunit subtype-selective compounds acting at the benzodiazepine site.

## 1. Introduction

Shortly after their development in the 1960s, benzodiazepines became very popular as they exerted many desirable effects such as reduction of anxiety, anticonvulsant properties, and myorelaxation combined with a rather low toxicity [1]. However, their use is associated with many side effects precluding their long-term use, including sedation, amnesia, cognitive impairment, and ataxia. Even though guidelines generally recommend limiting benzodiazepines to short-term use, long-term use still often occurs. Chronic benzodiazepine treatment can result in the development of benzodiazepine dependence [2]. DSM-IV criteria for benzodiazepine dependence consist of various psychological (behavioral) and physical symptoms, including tolerance, withdrawal symptoms when drug intake is stopped and dose escalation [3]. Indeed, chronically treated patients become less sensitive to some effects of benzodiazepines (tolerance) which may include anticonvulsant, sedative, hypnotic, and myorelaxant effects of benzodiazepines. Also, benzodiazepine discontinuation may result in the appearance of a characteristic withdrawal syndrome with heightened anxiety, insomnia, and sensory disturbances [4]. In fact, tolerance and withdrawal could be two manifestations of the same compensatory mechanism, with withdrawal occurring when the counterbalancing benzodiazepine effect is absent [5]. This is supported by the fact that acutely induced benzodiazepine effects are opposite to the withdrawal symptoms, and that changes in glucose use in the Papez circuit (including the cingulate cortex and mammillary body) were also observed on withdrawal, implying a common circuitry in the withdrawal process [6]. However, physical dependence (usually defined by withdrawal symptoms) does not require the presence of tolerance, and tolerance may develop without any signs of physical dependence [7].

Presently, despite decades of basic and clinical research, our understanding how benzodiazepines tend to lose their efficacy over time (i.e., tolerance) is at least incomplete. Here we review the current knowledge on the neuroadaptive mechanisms underlying benzodiazepine tolerance. This paper does not specifically address the addictive properties of benzodiazepines and their effects on the dopamine system or their abuse liability potential (including their nonmedical use in popular culture), which are described in detail elsewhere [8–10].

 Benzodiazepine tolerance is considered to constitute an adaptive mechanism following chronic treatment, and it may thus be regarded as an example of neuronal plasticity. Efforts have been made to explain tolerance at the molecular or functional level of the GABA_A_ receptor because classical (nonselective) benzodiazepines modulate inhibitory GABA_A_ receptors possessing *α*_1_, *α*_2_, *α*_3_, or *α*_5_ subunits. On the other hand, the excitatory glutamate system has also been implicated to play a role in the development of benzodiazepine tolerance [5]. Enhanced understanding of the dynamic process leading to reduced benzodiazepine efficacy following chronic treatment could accelerate the development of compounds that would maintain efficacy during chronic treatment [11]. Indeed, increasing knowledge on the specific functions of different GABA_A_ receptor subunits has led to a breakthrough of novel and more selective drugs acting at the benzodiazepine site of the GABA_A_ receptor. It is interesting but beyond the scope of the review to draw a comparison between benzodiazepine tolerance and alcohol tolerance as alcohol (albeit with low potency) acts at the GABA_A_ receptor [12].

Firstly, we will discuss the molecular basis of the GABA_A_ receptor system before taking a closer look at the clinical aspects of the development of benzodiazepine tolerance. Then, the putative molecular mechanisms underlying benzodiazepine tolerance will be extensively discussed, followed by a section specifically addressing the issue of tolerance development with novel and more selective benzodiazepines in the light of the putative tolerance mechanisms associated with classical benzodiazepines. From a clinical perspective, the understanding of tolerance is important because long-term benzodiazepine treatment with continuing efficacy—using either existing or novel and more selective drugs—could offer potential benefits to several groups of patients.

## 2. Benzodiazepines and the GABA_A_ System

### 2.1. GABA_A_ Receptors

**Figure 1:**
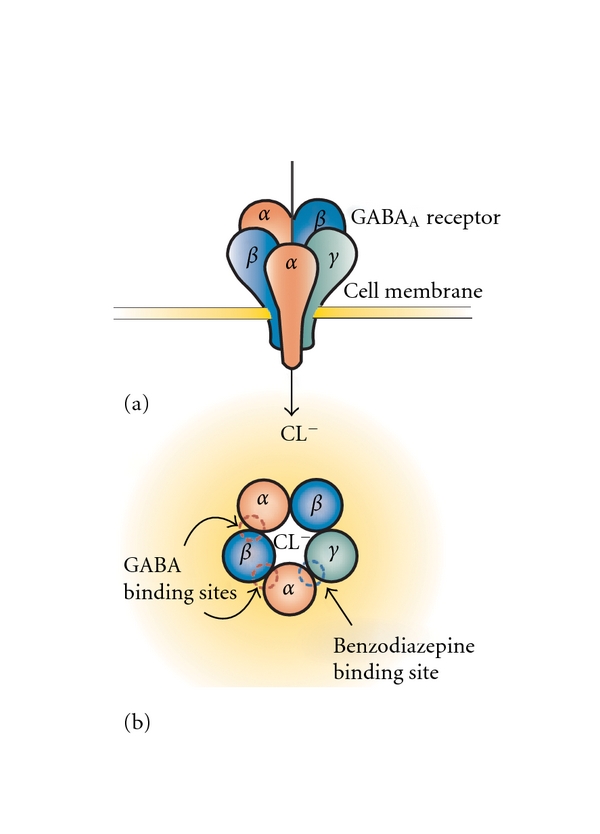
Representation of the GABA_A_ receptor structure. The inhibitory GABA_A_ receptor consists of five subunits that together form a ligand-gated chloride (Cl^−^) channel (a). When GABA binds (between the *α* and the *β* subunit of the GABA_A_ receptor), chloride ions flow into the neuron, resulting in a hyperpolarization of the cell membrane (a). Classical nonselective benzodiazepines allosterically enhance the inhibitory actions of GABA by binding between the *α*_1_, *α*_2_, *α*_3_, or *α*_5_ subunit and the *γ* subunit (b). Although the GABA_A_ receptor displays a large molecular heterogeneity depending on the subunit composition, the most common subtype is a pentamer with 2*α*, 2*β*, and 1*γ* subunit.

GABA_A_ receptors constitute the major fast inhibitory neurotransmitter system in the brain. They are composed of five transmembrane-spanning subunits that assemble to form a ligand-gated chloride channel with various possible subunits (*α*_1–6 _, *β*_1–3 _, *γ*_1–3 _, *δ*, *ε*, **θ**, and **π**) resulting in GABA_A_ receptor heterogeneity [13]. Binding of GABA to the GABA_A_ receptor increases the influx of negatively charged chloride ions, resulting in an inhibitory postsynaptic signal (IPSP). Although in theory a vast number of subunits combinations could be expected, GABA_A_ receptors are found in typical subunit compositions with the most common receptor subtype being composed of two *α*, two *β*, and one *γ* subunit [14] (Figure 1). In situ hybridization and immunohistochemical studies have shown that GABA_A_ receptor subunits display a distinct CNS distribution with a differential cellular localization pattern, suggesting that GABA_A_ receptor subunits have a specialized function (Table 1) [14]. Overall, a high expression of GABAergic subunits is present in the cortex, hippocampus, and basal ganglia [15]. Of the GABAergic subunits, *α*_1_, *β*_1_, *β*_2_, *β*_3_, and *γ*_2_ subunits are found throughout the brain. In contrast, the *α*_2_, *α*_3_, *α*_4_, *α*_5_, *α*_6_, *γ*_1_, and *δ* subunits have a specific regional expression pattern. The *α*_1_ subunit is highly coassembled with *β*_2_ and *γ*_2_ subunits and is synaptically located on neuronal cell bodies. GABA_A_ receptors that contain an *α*_2_ or *α*_3_ subunit are less abundant and are codistributed with the *β*_3_ and *γ*_2_ subunits. The *α*_2_ subunit is present in the cortex, hippocampus, amygdale, and hypothalamus, and often its expression is negatively correlated with the expression of *α*_1_ subunits. The expression of the *α*_3_ subunit is highest in the cortex, hippocampus, amygdala, thalamus, and brainstem, although it is also expressed in monoaminergic neurons (e.g., the raphe nuclei and the locus coeruleus in the brainstem) and cholinergic neurons in the forebrain. *α*_5_ subunits are predominantly expressed in the hippocampus where they comprise 15–20% of the diazepam-sensitive GABA_A_ receptors [16]. Regarding cellular localization, cortical and hippocampal pyramidal cells receive input from morphologically distinct GABAergic interneurons that innervate different pyramidal cell parts depending on the type of interneuron (e.g., chandelier and basket cells) with a specialized postsynaptic expression of *α* subunits [17, 18].

Thus, GABA_A_ receptor subtypes probably possess diverging functional properties dependent on the subunit composition, contributing to the GABA signaling complexity [13]. Additionally, GABA_A_ receptors are found synaptically as well as extrasynaptically. Synaptic receptors usually contain *γ* subunits and mediate fast phasic inhibition accompanied by transient high GABA concentrations [16]. By contrast, GABA has higher potency (at *μ*M concentrations) at extrasynaptic GABA_A_ receptors that usually contain a *δ* subunit, preferentially assemble with *α*_6_ or *α*_4 _subunits and have slow desensitization kinetics [20]. Also, *α*_5_ subunits may be localized extrasynaptically [21]. Extrasynaptic tonic inhibition—which is not modified by benzodiazepines—is suggested to modulate excitability of neuronal networks throughout the brain.

### 2.2. Benzodiazepines from a Nonspecific towards a Subunit-Specific Pharmacology

Classical benzodiazepines allosterically modulate GABA-induced IPSPs by binding to the benzodiazepine site of GABA_A_ receptors that contain an *α*_1_, *α*_2_, *α*_3_, or *α*_5_ subunit in combination with a *β* and a *γ*_2_ subunit (Figure 1). The exact binding site of benzodiazepines at the GABA_A_ receptor is located between the *α* and *γ* subunit. In contrast, benzodiazepines do not interact with GABA_A_ receptors that contain an *α*_4_- or *α*_6_-subunit. In addition to benzodiazepines, other drug classes can bind to the GABA_A_ receptor complex, including several anticonvulsants, ethanol, barbiturates, neurosteroids, and some anesthetics [15]. The fact that classical benzodiazepines non-selectively bind to different *α* subunits led to the hypothesis that the pharmacological profile with anxiolytic, sedative, anticonvulsive and myorelaxant properties may be further dissected. Both genetic and pharmacological approaches explored the hypothesis that *α* subunits differentially contribute to the different effects of classical benzodiazepines The genetic approach consisted of point mutations into specific *α* subunits (*α*_1_(H101R), *α*_2_(H101R), *α*_3_(H126R), and *α*_5_(H105R)), turning them functionally insensitive to benzodiazepines without altering their GABA sensitivity [22]. Pharmacological research on the GABA_A_ receptor has focused on the development of compounds that show differential efficacy across the various *α* subunits [13]. Such drugs generally bind with equal affinity to all *α* subunits (i.e., *α*_1_, *α*_2_, *α*_3_, and *α*_5_ subunits), but selectively alter the capacity to increase GABA binding to one or more of them. Using this strategy, various efficacy-selective (and some affinity-selective) compounds have been developed with preferential agonistic activity at the *α*_1_ (zolpidem and zaleplon), *α*_2/3_ (TPA023, L838, 417, and SL651498), or inverse agonistic activity the *α*_5_ subunit (*α*5IA, L-655,708, and MRK-016) (see also Table 2). 

In line with a specific central localization and distribution of GABAergic subunits, these genetic and pharmacological approaches have demonstrated that different *α* subunits of the GABA_A_ receptor mediate the distinct effects of benzodiazepines. Specifically, *α*_1_-containing GABA_A_ receptors probably mediate the sedative, amnesic, and anticonvulsant actions of classical benzodiazepines [13, 23]. In contrast, muscle relaxation and anxiety reduction after benzodiazepine administration was primarily ascribed to *α*_2_ (and possibly *α*_3_) subunit activation [24], whereas *α*_5_ subunit-containing GABA_A_ receptors appear to be involved in learning and memory [25, 26].

In light of the topic of this review, studies investigating the contribution of GABAergic subunits in benzodiazepine abuse liability, drug reinforcement, and tolerance development are of particular interest. Unfortunately, studies applying genetic and subtype-selective methodologies to examine the development of tolerance are scarce. One study using *α* subunit point mutation mice implicated a critical role for *α*_5_ subunits together with *α*_1_ subunits in the decreasing sedative efficacy of the classical benzodiazepine diazepam after chronic treatment [27]. We will discuss this finding in detail later in this paper. Studies on the background of physical dependence and abuse liability using subtype-selective GABA_A_R modulators are more abundant. Using self-administration studies, it was shown that efficacy at *α*_1_-containing GABA_A_ subtypes significantly contributed to the reinforcing effects and withdrawal symptoms of benzodiazepines [8, 28, 29]. Specifically, TPA123, which still possesses 23% intrinsic activity at *α*_1_ subunits still led to benzodiazepine-like drug reinforcement and withdrawal symptoms, whereas TPA023 with 0% *α*_1_ intrinsic activity did not, even at full GABA_A_ receptor-binding capacity [8]. However, there is still the possibility that the lower *α*_2_ and *α*_3_ efficacy of TPA023 may have contributed to the absence of drug reinforcement and withdrawal. In support, L-838,417 also led to continued self-administration, even though it lacks efficacy for the *α*_1_ subtype [30]. In any case, the *α*_5_ subunit may not be directly involved in the abuse potential of classical benzodiazepines as the *α*_1_-preferring hypnotic zolpidem with no affinity for the *α*_5_ subunit still led to self-administration in primates [30]. This finding is surprising as it suggests that the *α*_5_ subunit may be involved in tolerance development but not in drug reinforcement. Consequently, these processes could be independently mediated, even though they are both incorporated in the definition of benzodiazepine dependence.

### 2.3. GABA Metabolism

As benzodiazepines enhance the inhibitory effects of GABA and shift the GABA concentration-response curve to the left, the synaptic GABA concentration affects benzodiazepine efficacy. GABA is converted from glutamate by the enzyme glutamic acid decarboxylase (GAD) that maintains intracellular levels of GABA and exists in two independent isoforms (GAD_65_ and GAD_67_). In contrast to the localization of GAD_67_ in the neuronal body, GAD_65_ is primarily expressed in axon terminals, suggesting a role for GAD_65_ in synaptic neurotransmission and a more general role for GAD_67_ in regulating GABA synthesis [32]. Synaptic GABA is removed from the cleft into the presynaptic axon terminals by GABA transporters (GATs). So far, four GAT subtypes have been identified, with the highly expressed GAT_1_ and GAT_4_ being the most widely distributed [33].

## 3. The Development of Benzodiazepine Tolerance

Before examining the possible mechanisms underlying the development of benzodiazepine tolerance after long-term exposure, it is important to review its evidence and determine whether it is clinically relevant. Overall, there is little doubt that benzodiazepines are acutely effective in reducing anxiety, sleep latency and preventing convulsions. The tolerance that is eventually thought to develop appears to occur at different rates and to a different degree for each of the benzodiazepine effects [34]. Preclinical studies have shown that tolerance to the sedative and hypnotic effects occurs rather rapidly, followed by tolerance to the anticonvulsant effects, whereas tolerance to the anxiolytic effects of benzodiazepines are absent or partially develop after long-term treatment (for reviews, see [34–36]). As these preclinical studies have already been extensively reviewed, and novel preclinical studies on benzodiazepine tolerance have been limited in the last years to our knowledge, it is beyond this paper to reproduce all preclinical data on tolerance development. In general, preclinical studies are in agreement with the clinical divergent picture, even though in most preclinical studies, tolerance is not directly related to the applied dose, dosing interval, or the drug's plasma levels or half-life. Here, we will focus on the clinical evidence for (the rate of) tolerance development for each benzodiazepine action, even though we will also include preclinical studies when clinical studies are lacking or inconclusive.

### 3.1. Clinical Studies on Sedative and Hypnotic Tolerance

A study in low-dose benzodiazepine-dependent subjects showed a complete loss of hypnotic activity independent of the half-life of the prescribed benzodiazepine, even though a substantial suppression of REM sleep still occurred [37]. Also, other studies have shown that chronic users displayed no increase in sedation or motor impairment after the acute application of a benzodiazepine [38–40]. Moreover, tolerance to benzodiazepine-induced decreased reaction speed was shown after 10 days of alprazolam treatment [41]. Oral administration of triazolam, a short acting benzodiazepine, initially improved both sleep induction and maintenance, but latency to sleep and the number of awakenings were back to baseline values after two weeks of triazolam use [42]. Importantly, early-morning insomnia associated with short-acting benzodiazepines triazolam and midazolam markedly worsened after 7 days of treatment [43]. However, conflicting studies with triazolam exist that did not show any tolerance development [44, 45]. Another study applying the longer-acting benzodiazepine temazepam (15 or 30 mg) for either 26 or 54 nights in 7-8 subjects with chronic insomnia found no development of drug tolerance due to long-term temazepam administration [46]. Flurazepam, which has a relatively long elimination half-life, was shown to be effective for initiating and maintaining sleep with intermediate and long-term use (over 4 weeks), even though daytime sedation diminished during prolonged use [47]. Thus, even though tolerance to the sedative effects quickly emerges in most studies, these effects seem to be most prominent with benzodiazepines with a short half-life. Tolerance could thus depend on the half life of the applied benzodiazepine. However, this may be an overgeneralization, as a review showed that tolerance in human subjects only marginally emerged after chronic treatment with the short-acting drugs midazolam and zolpidem, even though the short-acting drug triazolam was associated with tolerance [48]. A limitation of most studies is their relatively short duration of exposure. Another issue is that convincing evidence for improved sleep after long-term use is lacking [49], yet this may not be the sole result of tolerance but could also be attributable to a generalized lack of efficacy. In support, in human subjects, discontinuation of benzodiazepines did not decrease sleep quality compared to a group that stayed on benzodiazepines up to 52 weeks after cessation [50], or even increased sleep quality and slow wave sleep after discontinuation in insomnia patients [51].

### 3.2. Clinical Studies on Anticonvulsant Tolerance

The use of benzodiazepines over a longer period of time in epilepsy is limited due to the development of tolerance [52]. In line with preclinical studies [53–55], tolerance develops during the first several months in 30–50% of epilepsy patients treated with either clobazam or clonazepam [56]. Thus, benzodiazepines are only prescribed in acute epileptic seizures or in a status epilepticus. However, in certain cases, intermittent use may be indicated, which may reduce the likelihood of tolerance [57]. Chronic treatment in rodents with the *α*_1_-preferential compound CL218, 572 resulted in loss of picrotoxin-induced seizures [58]. In contrast to classical benzodiazepines, partial GABA_A_ receptor PAMs including bretazenil did not result in anticonvulsant tolerance in several preclinical studies [54, 59, 60]. However, to our knowledge, these drugs have not been tested for (continuing) anticonvulsant activity in humans, precluding firm conclusions on their tolerance-inducing effects in epilepsy patients.

### 3.3. Clinical Studies on Amnesic Tolerance

Most studies have found continued short-term memory impairment after acute administration of benzodiazepines in chronically treated subjects [38, 39, 61]. Also, no tolerance for memory-impairing effects of alprazolam was found during a 10-day acute treatment [41]. However, another study reported tolerance to the acute amnesic effects of alprazolam after chronic use [40]. A major concern is that loss of memory associated with benzodiazepine use may be lasting, even after treatment discontinuation [62, 63], although other studies reported improved cognitive functioning after discontinuation with increased speed and accuracy of information processing, improved reaction time and working memory [50, 64–66]. Collectively, clinical data do not support the existence of tolerance to benzodiazepine-induced cognitive impairments.

### 3.4. Clinical Studies on Anxiolytic Tolerance

If developing al all, tolerance to the anxiolytic effects seems to develop more slowly compared to tolerance to the hypnotic effects. In patients with panic disorder, neither anxiolytic tolerance nor daily dose increase was observed after 8 weeks of alprazolam treatment with continued efficacy [67]. This was confirmed by another study in panic disorder patients who already chronically took alprazolam. Here, no differences were found in cortisol responsivity or anxiolytic efficacy compared to alprazolam-naïve patients, independent of disease severity [40]. Another double-blind study allocated 180 chronically anxious outpatients to diazepam (15 to 40 mg/day) and found that prolonged diazepam treatment (6–22 weeks) did not result in tolerance to the anxiolytic effects of diazepam [68]. Furthermore, additional studies all show a continuing anxiolytic effect, at least for panic disorder [69–72], generalized anxiety disorder [73], and social phobia [74–76]. Although a declining anxiolytic efficacy after long-term use of benzodiazepines cannot be clearly established, it is important to remember that other disadvantages prevent benzodiazepines to chronically treat anxiety symptoms, such as continued memory impairment, accident risk, hip fractures, and withdrawal symptoms [7, 77]. In conclusion, there is no solid evidence from the existing literature that anxiolytic efficacy declines following chronic benzodiazepine use in humans.

### 3.5. Clinical Studies on Drug Reinforcement Tolerance

The relevant topic of benzodiazepine tolerance to the reinforcing effects of benzodiazepines was already discussed by Licata and Rowlett [9]. They concluded that tolerance to reinforcing effects of benzodiazepines appears unlikely, supported by studies in nonhuman primates in which midazolam and zolpidem maintained stable self-injection and physical dependence under conditions of chronic continuous availability [78, 79]. Also, in humans, tolerance to drug reinforcement could lead to dose escalation that would maintain the vicious cycle of tolerance and dependence. In clinical practice, the majority of patients do not escalate their dose, suggesting that drug reinforcement tolerance may not emerge [80].

### 3.6. Conclusion

In conclusion, tolerance develops relatively quickly for the sedative, hypnotic, and anticonvulsant actions of benzodiazepines. Tolerance to anxiolytic and amnesic effects most probably does not appear at all. The fact that benzodiazepine dosage may be hard to reduce after chronic use can be ascribed to physical dependence to avoid withdrawal symptoms rather than the development of tolerance.

With diverging rates and varying completeness of tolerance development, it may be speculated that either (i) different tolerance mechanisms exist depending on the benzodiazepine effect, or that (ii) a uniform mechanism accounts for tolerance but revolves around the subunit composition of the targeted GABA_A_ receptor subtype and the brain region involved. However, from the presented evidence it is difficult to conclude that benzodiazepines indeed produce a robust and reproducible tolerance for all (side) effects. It is clear however, that benzodiazepine tolerance is not a uniform process for all clinical effects and does not apply to all available benzodiazepines. However, it is not known which factors predict whether a certain benzodiazepine possesses the potential to produce tolerance. Unfortunately, many studies address the physical dependence of benzodiazepines and their abuse potential, but do not specifically investigate tolerance.

## 4. Mechanisms Underlying Tolerance

### 4.1. General

Decades of research into the molecular effects of long-term benzodiazepine treatment have already importantly advanced our understanding of tolerance and several excellent reviews on this topic have already been published [5, 11, 34, 77]. The general assumption is that chronic benzodiazepine use leads to compensating changes in the central nervous system. This way, the GABA_A_ receptor may become less responsive to the continuing acute effects of benzodiazepines, either as a result of adaptations in the GABA_A_ receptor itself, intracellular mechanisms, or changes in other neurotransmitter systems, such as the glutamatergic system. Although adaptive processes probably play an important role, it is important to realize that the development of tolerance is not uniform for all its actions, and differences between preclinical and clinical tolerance development may exist. Therefore, the possibility that not one but multiple adaptive mechanisms simultaneously coexist complicates research into benzodiazepine tolerance. Moreover, these adaptive changes could be limited to one or more specific brain areas. This makes it very challenging to single out one a priori unifying mechanism underlying tolerance. In support, a study in rats using 2-deoxyglucose quantitative autoradiography showed that during chronic diazepam treatment, heterogeneous tolerance to the diazepam-induced reduction of glucose utilization occurred in the brain, depending on treatment duration and brain region [6]. Whereas acute diazepam administration resulted in reductions in glucose utilization throughout the brain, 3 days of diazepam treatment led to tolerance in brain structures associated with sensory processing (parietal cortex, auditory cortex, cochlear nucleus) which was interpreted to correlate with reduced sedation. After 28-day diazepam treatment, tolerance to the depressant effect of diazepam on cerebral glucose occurred in the mamillary body, subiculum, and caudate nucleus, whereas changes in the frontal cortex approached significance. Of particular interest is the finding that none of the amygdaloid nuclei showed any blunting over time, in line with persistent anxiolytic effects of benzodiazepines.

Before taking a closer look at specific mechanisms that have been proposed to underlie benzodiazepine tolerance, it is important to note that pharmacokinetic factors probably do not play a major role in the development of tolerance [81]. In support, plasma levels after acute diazepam administration did not differ between chronically alprazolam-treated and untreated panic disorder patients, even though sedative and amnesic tolerance was observed [40]. The most obvious candidate to mediate the adaptive changes in cellular and synaptic function after chronic benzodiazepine treatment is the GABA_A_ receptor. Therefore, we will first discuss the evidence supporting changes in the GABA system (including GABA_A_ receptor coupling and GABA receptor expression) after chronic benzodiazepine exposure.

### 4.2. GABA_A_ System Hypotheses

#### 4.2.1. Mechanism 1: GABA_A_ Receptor Uncoupling

One explanation for a loss of benzodiazepine function is a loss in GABA_A_ receptor allosteric coupling. The GABA_A_ receptor contains two GABA-binding sites and one benzodiazepine-binding site that are allosterically coupled, that is, binding to the benzodiazepine-binding site potentiates binding of GABA to the GABA-binding site (Figure 1). Benzodiazepines are generally referred to as positive allosteric modulators (PAMs) because their binding alters the GABA_A_ receptor conformation with an increased capacity to bind GABA, leading to increased channel opening frequency, increased chloride influx, and, consequently, to hyperpolarization. GABA_A_ receptor uncoupling is defined as a decreased ability of benzodiazepines to enhance GABA-induced IPSPs at the GABA_A_ receptor. In terms of tolerance development, it has been hypothesized that chronic treatment affects the benzodiazepines' capacity to pharmacologically enhance the GABA response (i.e., tolerance leads to uncoupling). A decreased coupling may develop as a result of changed GABA_A_ receptor subunit composition, alterations to the GABA_A_ receptor itself (including phosphorylation) or its second messenger ligands, or any process affecting the conformational state of the GABA_A_ receptor. The receptor uncoupling hypothesis is attractive as it does not assume any changes in subunit expression and ligand binding yet uses the knowledge on the specialized functions of the GABA_A_ receptor and the different subunits. However, the uncoupling process is an aspecific process as it can be induced by exposure to different classes of GABA_A_ receptor modulators acting at different modulatory sites, such as neurosteroids and barbiturates [82].

Already in 1984, an electrophysiological study indicated that allosteric coupling may play a role by showing a 50% decrease in the GABA enhancement of benzodiazepine-binding without significant changes in benzodiazepine-binding site density or affinity [83]. Also, more recent indications for reduced allosteric coupling were found after chronic treatment using transfected cell lines that express GABA_A_ receptors or in neurons [84–94]. The mechanisms underlying possible differences in coupling remain poorly understood. If the GABA_A_ receptor assembly process is modified, GABA receptor composition can be modified due to subunit replacements or altered expression in the receptor. This way, GABA_A_ receptors with a different functionality could potentially possess reduced benzodiazepine sensitivity due to reduced GABA_A_ receptor coupling. To our knowledge, no studies exist which have directly investigated GABA_A_ receptor subunit composition after chronic exposure. Another mechanism to affect receptor coupling is GABA_A_ receptor phosphorylation. GABA_A_ receptors are phosphorylated by various protein kinases and dephosphorylated by phosphatases [95]. Dynamic functional alterations in GABA_A_ receptor phosphorylation status may directly affect the inhibitory synaptic strength, with changes in channel openings (or indirectly influence receptor trafficking). However, the precise effects of phosphorylation on neuronal GABA_A_ receptor function are complex, even though key residues within the intracellular loop of the GABA_A_ receptor seem of particular importance. Using whole-cell patch-clamp recordings of GABA_A_ receptor IPSCs in hippocampal neurons, brain-region-dependent effects of activation of cAMP-dependent protein kinase A (PKA) or Ca^2+^/phospholipid-dependent protein kinase C (PKC) were shown [96]. Also, PKA activity was found to be directly involved in changed GABA_A_ receptor functioning in hippocampal pyramidal cells following chronic flurazepam treatment [97]. Probably, phosphorylation patterns rather than individual sites are of importance, supported by the finding that mutation to one PKA phosphorylation site is not involved in tolerance [90]. Using a point mutation genetic approach, transcriptional reduction was found in calcium-/calmodulin-dependent kinase II*α* and MAP kinase phosphatase-1in control mice but not in *α*1(H101R) after acute administration of diazepam [98]. Unfortunately, no chronic treatment was included in these studies.

It remains to be seen whether changes in allosteric coupling are relevant to the development of tolerance *in vivo*. Because benzodiazepine tolerance gradually develops over days to weeks, this would suggest that structural changes take place, whereas posttranslational compensation would be expected to be directly manifest. In support, uncoupling seems to develop rapidly, with the classical benzodiazepine chlordiazepoxide (applied together with GABA) stimulating the rate and extent of desensitization produced in a single neuron within several seconds [99]. Also, the observed uncoupling after chronic benzodiazepine treatment is rapidly reversed by a brief exposure *in vivo* to the benzodiazepine antagonist flumazenil [83, 86].

#### 4.2.2. Mechanism 2: Alterations in GABA_A_ Receptor Subunit Expression

The most straightforward hypothesis to explain impaired sensitivity after chronic benzodiazepine exposure would be a general downregulation of GABA_A_ receptors throughout the brain. Indeed, the process of tolerance requires GABA_A_ receptors at least to some extent, as cell lines expressing one specific type of the GABA_A_R are susceptible to tolerance [86, 87, 90]. Because classical (nonselective) benzodiazepines bind to GABA_A_ receptors that contain an *α*_1_, *α*_2_, *α*_3_, or *α*_5_ subunit, it could be expected that expression of receptors containing these *α* subunits (plus a *γ*_2_ subunit) is changed. Of course, this would depend on the cellular and anatomical distribution of GABA_A  _ receptors. Already earlier in Section 2.1, the differentiated and unique distribution of GABAergic subunits in the CNS was discussed. With regard to the benzodiazepine-sensitive *α* subunits, the *α*_1_ subunit is ubiquitously expressed in the entire brain, whereas the other *α* subunits (*α*_2_, *α*_3_ and *α*_5_) display a more restricted pattern of expression (see Table 1). If receptor internalization simply downregulates GABA_A_ receptor density, then a priori regional differentiation would be expected based on receptor distribution.

**Figure 2:**
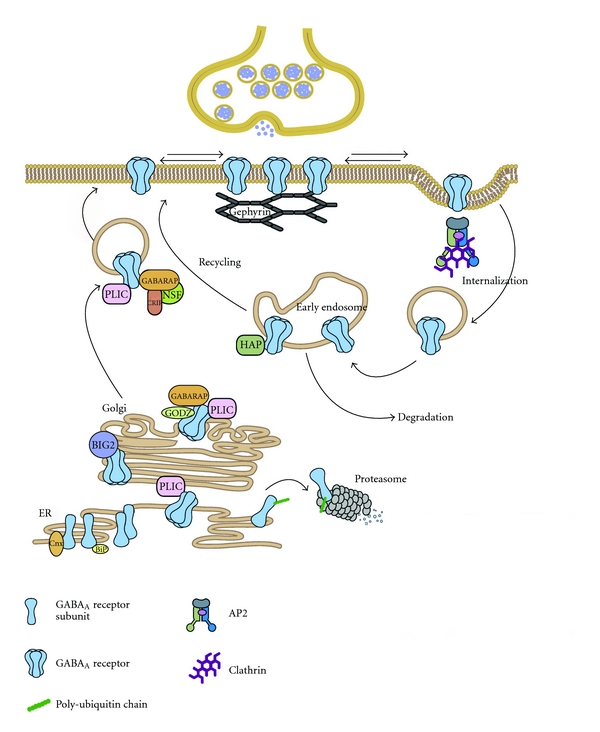
GABA_A_  receptor trafficking and associated proteins. GABA_A_ receptors are assembled from individual subunits in the endoplasmatic reticulum (ER) where the chaperones BiP and Calnexin assist in quality control. Unassembled GABA_A_ receptor subunits that are to be targeted for ER-associated degradation are ubiquitinated and degraded in the proteasome. The ubiquitin-like protein PLIC can interact with GABA_A_ receptors thereby inhibiting their targeting for proteasomal degradation. Assembled pentameric GABA_A_ receptors exit the ER and bind the guanidine exchange factor brefeldin-A-inhibited GDP/GTP exchange factor 2 (BIG2) in the Golgi. Here they also interact with the palmitoylase transferase GODZ and Gamma-aminobutyric acid receptor-associated protein (GABARAP). GABARAP interacts with the NEM sensitive fusion (NSF) protein, as does the GABA_A_ receptor *β* subunit, and this association may facilitate transport of the receptor complexes to the cell surface. GABA_A_ receptors are inserted at extrasynaptic sites and can diffuse along the plasma membrane in and out of synaptic domains. At synapses they are stabilized by an interaction with the scaffolding protein Gephyrin. The interaction of the GABA_A_ receptor intracellular loops with the *μ*2 subunit of the adaptin complex AP2 is important for GABA_A_ receptor internalization. GABA_A_ receptors are delivered by a clathrin-mediated pathway to early endosomes where they can be targeted for degradation in the lysosome or for recycling upon binding of Huntington-associated protein (HAP1). Reprinted by permission from Elsevier, reprinted from [101].

The processes that control the assembly, membrane trafficking, and synaptic accumulation of GABA_A_ receptors are complex (for review, see [100]). In short, GABA_A_ receptors are assembled from individual subunits out of the endoplasmic reticulum within minutes after their translation, with amino acid sequences in the N-terminus influencing the GABA_A_ receptor subtype (Figure 2). Then, receptor trafficking to the plasma membrane takes place, facilitated by diverse helper GABA_A_ receptor-associated proteins (among that GABARAP, BIG2, PRIP, gephyrin, and radixin). Ultimately, (clathrin-dependent) endocytosis occurs after receptor dephosphorylation, after which degradation or recycling may ensue (Figure 2). If prolonged activation of the GABA system leads to receptor downregulation, then this could be established by interfering at multiple steps of the dynamic GABA_A_ receptor life cycle. These include decreased subunit mRNA transcription, subunit degradation in the endoplasmic reticulum (e.g., by ubiquitylation), decreased expression of GABA_A_ receptor-associated helper proteins, and alterations in the endocytosis of specific GABA_A_ receptor subtypes. The finding that the protein synthesis inhibitor cycloheximide and the RNA synthesis inhibitor actinomycin D blocked the effects of chronic diazepam exposure in recombinant cells expressing GABA_A_ receptors indicates that GABA_A_ receptor synthesis is of at least some importance [87].

Up to now, a plethora of studies have tried to address whether chronic benzodiazepine treatment indeed affects GABA_A_ receptor expression (and thus benzodiazepine binding sites) using compounds with different subtype selectivity profiles at different doses and varying treatment duration. A recent excellent review summarized all data on the regulation of GABA_A_ receptor subunit after chronic benzodiazepine treatment that was mostly studied in rats [102]. It is beyond the scope of this review to repeat the meticulous work laid down in this paper. Of all subunits, *α*, *β*, and *γ* subunits have been mostly examined. This paper confirms that both for mRNA and protein subunit levels, the available evidence leads to a divergent and sometimes conflicting picture, although the majority of the studies essentially do not show any significant difference in subunit expression [102]. Furthermore, a lack of consistency appears for subunit changes in different specific brain areas. Moreover, the length and method of chronic treatment seem relevant since differences in GABA_A_ receptor subunit mRNA levels after chronic diazepam treatment in rats can depend on whether diazepam is administered as daily systemic injections or via osmotic minipumps [103]. Binding studies also generally report no changes in benzodiazepine binding after chronic treatment [92, 93, 104]. Together, GABA_A_ receptor expression (both mRNA and protein levels) is not consistently and robustly altered after various long-term treatment regimens. Thus, a general central downregulation or even consistent region-specific changes in GABA_A_ receptor expression after chronic benzodiazepine use are not supported by the literature. Even though methodological differences (e.g., treatment regimen, species, route of administration, and applied drug) may account for some conflicting findings, the results seem overall inconsistent. Moreover, molecular results are often not combined with behavioral tests, preventing a direct correlation between behavioral tolerance and molecular changes. Clinical studies applying *in vivo* binding or postmortem GABA_A_ receptor expression after chronic benzodiazepine treatment are to the knowledge of the authors lacking.

Changes in rates of GABA_A_ receptor endocytosis, receptor membrane insertion, intracellular trafficking, and association with helper GABA_A_ receptor-associated proteins could still play a role, leading to a reduction in membrane surface receptors without affecting overall subunit protein expression (e.g., see [105]). Another interesting suggestion is that a possible loss of synaptic function after chronic exposure could be due to a shift to a perisynaptic or even an extrasynaptic localization of GABA_A_ receptors, away from clustering of GABA_A_ receptors at synapses (Figure 2) [106]. At least in alcohol research, such dynamic changes in plasticity at inhibitory synapses have been shown [107]. Moreover, it cannot be excluded that particular subunits play a role in the development of tolerance after chronic treatment in the absence a direct up- or downregulation. Using the previously mentioned *α* subunit point mutation mice, acutely administered diazepam still reduced locomotor activity in *α*5 (H105R) mice even after chronic 8-day diazepam treatment at a combined daily dose of 15 mg/kg [27]. This suggests that the *α*_1_ subunit that mediates the sedative effects remains responsive, indicating that simultaneous activation of the *α*_1_ and *α*_5_ subunit may be necessary for tolerance to the locomotor-reducing effects of classical benzodiazepines. Specifically, it was hypothesized that increased phasic signaling would alter extrasynaptic tonic inhibition mediated by *α*_5_-containing GABA_A_ receptors, whereas a decrease in hippocampal *α*_5_-specific binding was reported in diazepam-tolerant mice. Also, in contrast to *α*_1_-, *α*_2_-, and *α*_3_-containing receptors, *α*_5_-containing GABA_A_ receptors are located extrasynaptically at the base of dendritic spines where they can modulate excitatory glutamatergic input. However, *α*1(H101R) mice are not sensitive to the acute sedative benzodiazepine effects, making a comparison to isolated *α*_1_ subunit activation not possible. Moreover, only tolerance to the sedative effects of diazepam was reported. Thus, it may still be possible that tolerance to other benzodiazepine effects is mediated by other subunits. 

### 4.3. Glutamate System Hypotheses

#### 4.3.1. General

From the previous sections, we conclude that compensatory changes solely arising from the GABA system may at most partially explain the tolerance arising following chronic treatment with benzodiazepines. Glutamate is an excitatory neurotransmitter acting on glutamate receptors. Together with the GABA system, they constitute the two fast-acting and opposing neurotransmitter systems that can modulate synaptic plasticity. In support, close neuroanatomical connections exist between GABAergic and glutamatergic neurons [108, 109]. With a presence in at least 30–50% of all synapses in the CNS, inhibitory GABA and excitatory glutamate together coordinate the balance in the brain's excitability. Therefore, it is not surprising that as these two opposing and fast-acting neurotransmitter systems form a delicate balance, chronic (increased) activation of the GABAergic system during benzodiazepine treatment may pertubate glutamatergic transmission. The basis of benzodiazepine tolerance could then lie in sensitization of the glutamatergic system—a putative process that could account for the withdrawal symptoms after chronic benzodiazepine discontinuation [5, 110]. Such sensitization is reminiscent to adaptive glutamatergic processes as seen in kindling experiments, although it should be noted that kindling only occurs with intermittent and not after continuous treatment [111]. Glutamatergic sensitization could thus play a role in the development of tolerance as well as withdrawal symptoms upon cessation of treatment. Glutamatergic changes after benzodiazepine withdrawal will not be discussed here, but there are indications that the glutamatergic system plays a role in withdrawal states with accompanying increases in anxiety and seizure activity (for review see [5]). However, glutamate receptor mRNA and protein changes may be dynamic during withdrawal, with unchanged levels during the early phase of withdrawal but changes occurring several days later [112]. This consequently complicates the interpretation of withdrawal studies and their significance for our understanding of benzodiazepine tolerance.

Similar to the GABAergic system, the glutamate system is diverse and complex, generally being divided into ionotropic and metabotropic receptor types. Ionotropic glutamate receptors form a class of heteromeric ligand-gated cation channels that potentiate the influx of K^+^, Na^+^, or Ca^2+^ ions following glutamate binding. Three classes of the ionotropic glutamate receptor occur in het central nervous system: the NMDA receptor (N-methyl-D-aspartate), the AMPA receptor (alpha-amino-3-hydroxy-5-methyl-4-isoxazole-4-propionic acid), and the kainate receptor (for a recent review see [113]). Functional NMDA receptors contain two obligatory GluN_1_ and two regulatory GluN_2/3_ subunits and are vital for synaptic plasticity (for review, see [114]). Each GluN subunit contains extracellular loops where coagonists glycine or D-serine (GluN_1_ and GluN_3_ subunits) and glutamate (GluN_2_ subunits) can bind [115]. Although the channel is blocked by Mg^2+^ ions, changes in membrane potential can make the channel permeable to Na^+^, Ca^2+^, and K^+^ ions. The central distribution of GluN_2_ subunits eventually ensures heterogeneity in the NMDA receptor system. AMPA receptors are widespread heterotetrameric ligand-gated ion channels composed of four types of subunits (GluR_1–4_), and are crucial to long-term synaptic plasticity such as long-term potentiation (for review see [116]). Although glutamate possesses lower affinity for the AMPA receptor compared to NMDA receptors, faster excitation-inducing kinetics are present at the AMPA receptor. Relevant to this review, a study showed that AMPA receptor desensitization was caused by a rupture of a domain interface which allowed the ion channel to close, providing a simple yet elegant explanation [117]. Kainate receptors are made up of four subunits, GluR_5_, GluR_6_, GluR_7_, KA_1_, and KA_2_, which are similar to AMPA and NMDA receptor subunits and can be arranged in different ways to form a functional tetramer (for review, see [118]). Compared to NMDA and AMPA receptors, synaptic kainate receptors exhibit slow rise and decay properties.

#### 4.3.2. Mechanism 3: Role of Ionotropic Glutamatergic Receptors

Several studies have addressed the compensatory glutamate sensitization hypothesis during chronic benzodiazepine exposure to account for the development of tolerance (as reviewed by [5, 110]).

In rodents, the development of tolerance to the sedative effects of the classical benzodiazepines diazepam and chlordiazepoxide was prevented by coadministration of the NMDA receptor antagonists CPP, dizocilpine, MK-801, and ketamine [119–121]. Also, lorazepam-induced tolerance to its acute anticonvulsant effects was partially prevented with simultaneous CPP treatment [122]. In contrast, the development of tolerance to the anxiolytic effects of diazepam in a social interaction test was not blocked by concomitant administration of dizocilpine [123]. This suggests that the mechanism underlying tolerance to the anxiolytic effects of diazepam is different from that underlying tolerance to the sedative effects. Increases in cortical mRNA of NMDA NR_1_ and NR_2B_ subunits have been reported in rats tolerant to diazepam [124, 125], which were prevented by concomitant treatment with the NMDA receptor antagonist MK-801 [126]. However, another study showed decreases in hippocampal NR_2B_ subunits after chronic flurazepam treatment, even though the total amount of NMDA receptors was unchanged [127].

In support, after long-term (but not acute) lorazepam treatment, no differences were found in the affinity or density of NMDA receptors, even though increased *in vitro* glutamate release and NMDA-induced cGMP efflux in the hippocampus was reported [128]. Together, these data suggest that NMDA-dependent mechanisms contribute to the development of benzodiazepine tolerance. However, as anxiolytic tolerance was not blocked by NMDA receptor antagonism, the NMDA system could also play a differential role in tolerance depending on the specific behavioral effects [123]. Moreover, a straightforward glutamate sensitization may be an oversimplification, as tolerance to the sedative effects of lorazepam after 21-day treatment correlated with a decreased rather than an increased sensitivity for glutamate (using^[(3)H]^  glutamate binding) [129].

Even though the AMPA receptor antagonist GYKI 52466 did not affect the development of tolerance to the sedative effects of diazepam [121], changes in AMPA receptor subunits have been reported to be altered after long-term benzodiazepine exposure [130]. Specifically, significant reductions of mGLuR1 (cortex and amygdala) and mGluR2 mRNA (amygdala) were reported in rats treated chronically with diazepam, even though the effects were complex and dependent on treatment route (subcutaneous or intraperitoneal injections). Adding to the complexity of the published data, another study did not show changes in hippocampal GluR1-3 subunit proteins following chronic flurazepam treatment, even though mEPSCs were found and nonspecific binding was increased using the AMPA receptor antagonist^[(3)H]^  Ro48-8587 [131]. A genetic approach with GluR_1_ knockout mice showed that after subchronic flurazepam treatment, these mice developed a reduced and incomplete tolerance to the muscle relaxation and sedative effects of flurazepam, even though acute flurazepam effects were comparable between knockout and wild-type mice [132].

With regard to glutamatergic kainate receptors, we found no pharmacological or genetic studies investigating the development of tolerance.

 Together, the evidence does not support a universal and replicable glutamatergic component, even though there are indications that NMDA receptor blockade can prevent tolerance to at least some behavioral benzodiazepine effects. However, molecular data are diverse and sometimes inconsistent, which are reminiscent of the molecular changes in the GABA system after chronic benzodiazepine treatment (see Section 4.2.2).

### 4.4. Other Mechanisms

#### 4.4.1. Mechanism 4: Transcriptional and Neurotrophic Factors

Although the hypothesis that downstream signaling events adjust in response to chronic exposure to benzodiazepines seems plausible, a surprising paucity of data exist in this field. It is tempting to speculate on the expression of diverse helper GABA_A_ receptor-associated proteins (including GABARAP, BIG2, PRIP, gephyrin, and radixin) after long-term benzodiazepine use (Figure 2). In addition, changes in intracellularly located cAMP-response-element-binding protein (CREB) or calcium, vital in various second messenger systems, could be altered, and prolonged GABA concentrations in a neuronal culture have been shown to affect voltage-gated calcium channels [133]. However, until further studies provide additional proof for chronic benzodiazepine-induced downstream intracellular changes, the evidence that this process plays a role is inconclusive.

Neurotrophic proteins support neuronal survival, synaptic growth, and differentiation throughout the brain via tyrosine kinase receptors (Trk) and, with lower affinity, via p75 receptors (p75NTRs) [134]. Neurotrophic factors that have discovered so far include brain-derived neurotrophic factor (BDNF), neurotrophin-3 (NT-3) and neurotrophin-4 (NT-4), and nerve growth factor (NGF). Since they act as potent factors in regulating fast synaptic inhibition, adaptations leading to tolerance following chronic benzodiazepine treatment could in part be mediated via these neurotrophic factors. In support, BDNF (and NT-4) was found to acutely reduce postsynaptic GABA_A_ receptor immunoreactivity via activation of TrkB receptors [135–139], even though one study reported an increase [140], and another study reports that chronic BDNF treatment potentiates GABAergic inhibition [141]. This reduced immunoreactivity was hypothesized to be caused by a reduction in GABA_A_ receptor surface expression and was accompanied by reduced postsynaptic responses with the direct GABA_A_ receptor agonist muscimol [142]. Mechanistically, BDNF-induced suppression of GABAergic signaling was hypothesized to stem from altered GABA_A_ receptor composition, increased GABA_A_ receptor phosphorylation, decreased subunit synthesis, or increased postsynaptic receptor internalization or diffusion [139]. Interestingly, all these proposed mechanisms were already discussed in this paper. Thus, neurotrophin-induced changes may not be an independent mechanism, but be a player in a causal chain of events. Again, to our knowledge, no studies exist on the effects of chronic benzodiazepine treatment on neurotrophic expression and functionality.

#### 4.4.2. Mechanism 5: Serotonin, Dopamine, Acetylcholine Systems

**Figure 3:**
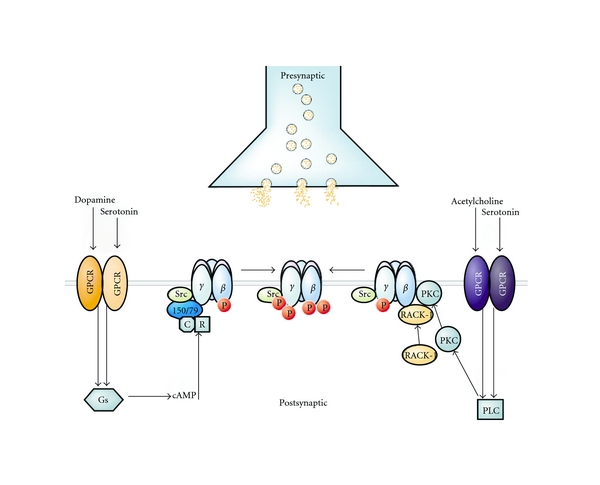
Functional crosstalk between G-protein coupled receptors (GPCRs) (which are present in the serotonin, dopamine, acetylcholine system) and GABA_A_ receptors is facilitated through multiple protein kinases and scaffold proteins. GABA_A_ receptor *β* and *γ*2 subunits are phosphorylated (P) by PKA and PKC upon the activation of individual GPCRs for dopamine and serotonin. PKA phosphorylation of GABA_A_ receptor *β*1 and *β*3 subunits is dependent upon AKAP150/79, which directly interacts with these receptor subunits. AKAP150/79 also binds inactive PKA composed of regulatory (R) and catalytic (C) subunits. In addition, PKC phosphorylates the receptor *β*1–3 and *γ*2 subunits. Upon the activation of the appropriate GPCR, PKC-mediated phosphorylation is facilitated by the direct (but independent) interaction of the receptor for activated C kinase (RACK-1) and the *β* isoform of PKC with the GABA_A_ receptor *β*1–3 subunits. RACK-1 facilitates functional regulation of GABA_A_ receptors by controlling the activity of PKC associated with these proteins. The GABA_A_ receptor *γ*2 subunit is also phosphorylated by Src, and this kinase is capable of binding to receptor *β* and *γ*2 subunits. Finally, the functional effects of phosphorylation are diverse and range from inhibitions to enhancements of GABA_A_ receptor activity, dependent upon the receptor subunit composition. Reprinted by permission from Elsevier, reprinted from [95].

There is ample evidence that the serotonin, dopamine, and acetylcholine receptor systems can modulate the GABA_A_ receptor functionality [143–146] (Figure 3). For example, the receptor for activated C kinase (RACK-1) potentiated PKC-dependent phosphorylation of GABA_A_ receptors mediated by the activation of muscarinic acetylcholine receptors [145], and serotonergic neurotransmission inhibited GABAergic signaling via GABA_A_ receptor PKC-dependent phosphorylation, again with involvement of RACK-1 [144]. Altogether, these neurotransmitter systems act via G-protein-coupled receptors to activate protein kinases (PKA and PKC) and scaffold proteins that may subsequently modulate GABA_A_ receptor *β* and *γ*_2_ subunit phosphorylation (Figure 3) [95].

However, studies investigating the role of the serotonin, dopamine, and acetylcholine system in response to chronic benzodiazepine treatment are scarce. Three weeks of diazepam treatment (25 mg/day) in healthy male volunteers resulted in tolerance to the prolactin and growth hormone response induced by the 5-HT precursor L-tryptophan, even though sedative effects of L-tryptophan remained present [147]. Another study showed that chronic diazepam treatment resulted not only in diazepam tolerance but also in a very modest reduced efficacy of the 5-HT_1A_ receptor agonist 8-OH-DPAT to induce flat body posture and forepaw treading [148]. In contrast, only acute but not chronic diazepam treatment decreased basal extracellular dopamine levels in rats, even though both acute and chronic treatment regimens could reverse the stress-induced rise of cortical dopamine levels [149].

#### 4.4.3. Mechanism 6: Neurosteroids

There is ample and convincing evidence that neurosteroids are endogenous allosteric regulators that interact with GABA_A_ receptors to modulate both tonic (extrasynaptic) and phasic (synaptic) inhibition (for reviews, see [150, 151]). Also, acute or chronic neurosteroid treatment may change GABA_A_ receptor subunit expression, especially extrasynaptic *α*_4_ and *δ* subunits [151]. In light of the plasticity-inducing actions of neurosteroids on inhibitory signaling, long-term enhancement of the GABA system with benzodiazepines may in turn evoke changes in the neurosteroids system such as changes in neurosteroid synthesis and metabolism, although classical benzodiazepines may differ in their potency to cause such changes [152]. In support, ovariectomy attenuated the development of tolerance to the anticonvulsant actions of diazepam [153]. Moreover, co-administration of the neurosteroids allopregnanolone or pregnenolone (but not dehydroepiandrosterone) prevented the development of tolerance after chronic treatment with either triazolam and diazepam [154]. Adding to the complexity of the putative involvement of neurosteroids in benzodiazepine tolerance, factors such as GABA_A_ receptor subunit composition, phosphorylation mechanisms, and ((extra)synaptic) localization—which are all factors that were already found to be involved in tolerance development—influence the specific dynamics of neurosteroid activity.

#### 4.4.4. Conclusion

From our review of the literature on the various mechanisms that may underlie benzodiazepine tolerance, it occurs that there is a considerable variance in the published data. The heterogeneity of the data lies in the application of different methodologies, species, treatment regimens, and benzodiazepines. Specifically, we have considered classical benzodiazepines as a homogenous drug class since they all lead to a nonspecific enhancement of GABA_A_ receptors that contain an *α*_1_, *α*_2_, *α*_3_, or *α*_5_ subunit. However, *in vivo* pharmacodynamic potency and pharmacokinetic half-life differences could greatly impact on tolerance processes [7]. In support, subchronic treatment with different classical benzodiazepines lead to differential propensity for FG7142-induced seizures in mice, with triazolam, clonazepam, and diazepam producing around seizures in around 80% of the mice, whereas alprazolam and midazolam did so in 60% of the animals and lorazepam in 40% of the animals [155]. Surprisingly, chlordiazepoxide did not lead to any precipitated seizures, even though a comparable GABA_A_ receptor occupancy was obtained. Therefore, the assumption that classical benzodiazepines act as a homogeneous class probably complicates the interpretation of the current literature.

Altogether, it appears that none of the proposed putative mechanisms can sufficiently explain tolerance development. Thus, multiple mechanisms may (synergistically) coexist, or an additional yet undiscovered mechanism may be present. However, the complex and adaptive nature of the GABA system and the existing heterogeneous literature on benzodiazepine tolerance suggest that one unifying tolerance mechanism may be a vast oversimplication. In any case, the proposed tolerance mechanisms are not completely independent, exemplified by the fact that neurotrophic factors and neurosteroids are influenced by GABA_A_ receptor composition and phosphorylation status, which are themselves proposed to be involved in benzodiazepine tolerance. Unfortunately, the present literature does not consistently support a clear recommendation in terms of a pharmacological GABA_A_ receptor profile (e.g., subunit preference) to aid in the development of novel and more selective benzodiazepines that lack tolerance development and are suitable for long-term treatment.

## 5. Tolerance to Novel Subtype-Selective Benzodiazepines

Here, we will review the evidence for tolerance development with novel GABA_A_ receptor subtype selective compounds that provide the direct opportunity to evaluate their roles in tolerance. With the development of subunit-selective benzodiazepines, it has become possible to dissect the different effects of classical benzodiazepines (see Section 2.2 and Table 2). However, declining efficacy over time is a complex process which may not be easily attributed to one specific *α* subunit. Still, if novel drugs possess reduced propensity to lead to tolerance development, this will be greatly welcomed from a clinical perspective. Continuing efficacy with these drugs would advance the clinical use of drugs acting at the GABA_A_ receptor benzodiazepine site. Unfortunately, not many studies have directly addressed tolerance development using these novel compounds. Recent data from our laboratory suggest that no tolerance develops to the acute hypothermic, anxiolytic, or sedative effect of diazepam in mice treated for 28 days with the GABA_A_-*α*_2_/*α*_3_ selective compound TPA023 (Table 2) [156], indicating that chronic activation of GABA_A_-*α*_2_/*α*_3_ receptors does not lead to anxiolytic tolerance after acute diazepam challenge (unpublished data). Also, in contrast to morphine, no analgesic tolerance occurred in rats after a 9-day treatment with the *α*_2/3_ subtype GABA_A_ receptor positive allosteric modulator L838,417 using a model of neuropathic pain [157]. From these data, it seems that tolerance development after chronic administration of GABA_A_-*α*_2_/*α*_3_ subtype selective drugs may not develop, or, alternatively, that tolerance to diazepam's sedative actions needs concomitant activation of GABA_A_-*α*_1_/GABA_A_-*α*_5_ receptors. In support of the latter hypothesis, ligands that do not bind to the *α*_5_ subunit such as zolpidem have a reduced tendency to engender tolerance [158, 159], supported by studies in which chronic treatment with zolpidem (but not midazolam) did not produce any tolerance to sedative and anticonvulsant effects in mice and rats [160–162]. 

In addition to studies directly assessing tolerance, several studies have investigated the precipitated withdrawal after (sub) chronic treatment with subtype-selective compounds. Compounds with selective efficacy at *α*_2_, *α*_3_, and *α*_5_  GABA_A_ receptor subtypes were shown to lead to differential seizures susceptibility in mice in response to the inverse agonist FG-7142 [155]. Chronic treatment with zolpidem, as well as the selective compounds L-838,417 (partial agonist at *α*2 GABA_A_, *α*3GABA_A_, and *α*5GABA_A_ receptors) and SL651498 (full agonist at *α*2GABA_A_ and *α*3GABA_A_ receptors, partial agonist at *α*1GABA_A_ and *α*5GABA_A_ receptors), did not result in seizures following FG-7142 administration [31, 155] (Table 2). Similarly, chronic treatment with TPA023 (partial agonist at *α*2GABA_A_, *α*3GABA_A_, and *α*5GABA_A_ receptors) also did not result in FG-7142-induced seizures in mice [156]. However, because these studies do not specifically address tolerance development, the rather general conclusion from these studies is that partial or selective modulation of the GABA_A_ receptor results in a reduced liability for physical dependence. Thus, it is important to note that, even though zolpidem does not seem to engender any obvious tolerance development, zolpidem can lead to withdrawal symptoms that are comparable to those seen after chronic classical benzodiazepine treatment [29, 77]. Thus, tolerance and withdrawal symptoms may constitute separate entities in benzodiazepine dependence. In support, one study demonstrated that marked withdrawal symptoms appeared upon abrupt discontinuation of chronic clorazepate treatment in dogs, even though tolerance was present to a rather limited extent [163].

Together, it can be concluded that so far, *α*_2_/*α*_3_ subtype selective compounds have neither been found to lead to tolerance nor withdrawal symptoms. This would constitute a significant improvement over currently used benzodiazepines, even though the anxiolytic profile of these compounds remains to be determined [164], and abuse liability may still be present [8]. However, interpretations should be made with caution since chronic treatment with nonselective partial positive allosteric modulators such as bretazenil did neither result in anticonvulsant tolerance [54, 59, 60] nor in FG-7142-precipitated seizures [155]. These studies implicate that the potency of classical and subtype-selective compounds, in addition to or despite subtype selectivity, may also be of importance in the development of tolerance. It could be also hypothesized that low efficacy at the *α*_1_ subunit, rather than selectivity or reduced efficacy at *α*_2_/*α*_3_ subtypes, may be the causal mechanism preventing tolerance development. Also, the clinical anxiolytic efficacy of *α*_2_/*α*_3_ subtype selective compounds has not yet been established. In addition to a specific efficacy profile, tolerance development may also depend on a compound's *affinity* at certain GABA_A_ receptor subtypes. This way, tolerance processes may be different with affinity-selective compounds such as zolpidem compared to efficacy-selective compounds such as TPA023. Circumstantial evidence stems from the fact that *α*_1_-preferential affinity-selective compounds such as zolpidem produce physical dependence [165], even though the compound TPA123 that possesses 23% efficacy at the *α*_1_ subunit (but is not affinity selective) did also result in physical dependence [8]. However, based on the currently available evidence, no definite conclusions can be drawn regarding the subtype involved in tolerance. Also, it is not possible to distinguish tolerance processes in selective binding (affinity) and selective activation (efficacy).

## 6. Conclusion

In the present paper, we summarized the rather inconsistent data regarding changes in several neurotransmitter systems to explain the development of tolerance. Specifically, we addressed possible changes at the level of (i) the GABA_A_ receptor (subunit expression and receptor coupling), (ii) intracellular changes stemming from transcriptional and neurotrophic factors, (iii) ionotropic glutamate receptors, (iv) other neurotransmitters (serotonin, dopamine, and acetylcholine systems), and (v) the neurosteroid system. From the large variance in the studies, it appears that either different (simultaneous) tolerance mechanisms occur depending on the benzodiazepine effect, or that one tolerance-inducing mechanism depends on the activated GABA_A_ receptor subtypes. This is not unlikely, given that tolerance is a heterogeneous process that occurs at different rates for the various effects and also depends on the profile of the (subtype selective) benzodiazepine. Adaptations could then occur on different time scales depending on the receptor subtype and brain region involved. In line with this hypothesis, tolerance develops relatively quickly for the sedative and anticonvulsant actions of benzodiazepines, whereas tolerance to anxiolytic and amnesic effects most probably do not develop at all. It is intriguing that anxiolytic effects of classical benzodiazepines may not decline during prolonged treatment. In addition to subtype selectivity, additional factors may be important for a (subtype-selective) benzodiazepine to cause tolerance, including GABA_A_ receptor potency (efficacy) and *in vivo* receptor occupancy over time. The finding that partial agonists with an overall but comparable lower efficacy at all *α* subunits of the GABA_A_ receptor such as bretazenil did not result in anticonvulsant tolerance raises the possibility that chronic clinical use of these compounds is associated with a lower tolerance.

An important question is how the development of tolerance of benzodiazepines could be reduced. One interesting suggestion could be—rather than intermittent use that can be defined by an individual—to develop benzodiazepine dosing schedules with varying daily doses including placebos. This could result in continued clinical efficacy (obviously depending on the indication) and utilize the placebo effect. The other possibility to reduce tolerance is the currently developing and promising body of literature on subtype-selective GABA_A_ receptor PAMs. From the literature we reviewed, it appears that *α*_2_/*α*_3_ subtype selective compounds do not lead to tolerance or withdrawal symptoms. However, the underlying mechanism (reduced *α*_1_ efficacy or a generally reduced efficacy profile) is unknown. Also, it is presently unclear whether this lack of tolerance also applies to *α*_1_- and *α*_5_-selective GABAergic positive allosteric modulators, although a broad and unspecific tolerance resulting from selective (and often low potency) compounds seems unlikely.

In conclusion, the development of tolerance following chronic benzodiazepine treatment is a complex process in which multiple processes may simultaneously act to cause varying rates of tolerance depending on the studied effect and the administered drug. There is no convincing evidence that subtype-selective compounds acting at the benzodiazepine site lead to tolerance at a level comparable to classical benzodiazepines. If this is indeed the case, one consequence may be that such subtype-selective compounds are unlikely to engender clinical tolerance, which would be a clinically significant improvement over classical benzodiazepines.

## Figures and Tables

**Table 1:** Localization of common GABA_A_ receptor subtypes in the brain (adapted from [19]).

Subtype	Frequency	Localization
*α*_1_ *β*_2_ *γ*_2_	Major (60%) synaptic	Cerebral cortex (layer I–VI), hippocampus, striatum, cerebellum, amygdala, brainstem.
*α*_2_ *β*_n_ *γ*_2_	Minor (15–20%) synaptic	Cerebral cortex (layers I–IV), hippocampus, striatum, hypothalamus, amygdala.
*α*_3_ *β*_n_ *γ*_2_	Minor (10–15%) synaptic	Cerebral cortex (layers V-VI), hippocampus, cerebellum, amygdala, brainstem (including raphe nuclei and locus coeruleus), spinal cord.
*α*_4_ *β*_n_ *δ*/*γ*	Minor (<10%) extrasynaptic	Hippocampus (dentate gyrus), thalamus, cortex.
*α*_5_ *β*_n_ *γ*_2_	Minor (<5%) extrasynaptic	Cerebral cortex, hippocampus, amygdala, hypothalamus, spinal cord.
*α*_6_ *β*_n_ *γ*2/*δ*	Minor (<5%)(extra) synaptic	Cerebellum.

**Table 2:** Summary of novel GABA_A_ receptor subtype selective compounds.

Target	Name	Efficacy (compared to a classical benzodiazepine)	Affinity/Remarks	Ref
*α*_1_	Zolpidem	Comparable at *α*_1_/*α*_2_/*α*_3_/*α*_5_	5-10-fold higher affinity for *α*_1_ versus *α*_2/3 _> 1000 fold higher affinity for *α*_1_ versus *α*_5_	[15]
*α*_2/3_	TPA023	*α*_1_ (0%), *α*_2_ (11%), *α*_3_ (21%), *α*_5_ (5%)	Equivalent affinity	[8]
*α*_2/3_	TPA123	*α*_1_ (23%), *α*_2_ (35%), *α*_3_ (43%), *α*_5_ (19%)	Equivalent affinity. Reinforcing efficacy and physiological dependence remained present	[8]
*α*_2/3_	L838,417	*α*_1_ (1.5%), *α*_2_ (43%), *α*_3_ (43%), *α*_5_ (39%)	Equivalent affinity	[23]
*α*_2/3_	SL651498	*α*_1_ (45%), *α*_2_ (115%), *α*_3_ (83%), *α*_5_ (50%) Compared to zolpidem for *α*_1_ efficacy	5–10-fold increased affinity for *α*_2/3_, 10–20 fold lower affinity for *α*_5_	[31]
